# Causal Discovery in Radiographic Markers of Knee Osteoarthritis and Prediction for Knee Osteoarthritis Severity With Attention–Long Short-Term Memory

**DOI:** 10.3389/fpubh.2020.604654

**Published:** 2020-12-18

**Authors:** Yanfei Wang, Lei You, Jacqueline Chyr, Lan Lan, Weiling Zhao, Yujia Zhou, Hua Xu, Philip Noble, Xiaobo Zhou

**Affiliations:** ^1^School of Biomedical Informatics, University of Texas Health Science Center at Houston, Houston, TX, United States; ^2^McGovern Medical School, University of Texas Health Science Center at Houston, Houston, TX, United States

**Keywords:** LSTM – Long Short-Term Memory, attention-LSTM, causal inference, prediction model, disease progression

## Abstract

The goal of this study is to build a prognostic model to predict the severity of radiographic knee osteoarthritis (KOA) and to identify long-term disease progression risk factors for early intervention and treatment. We designed a long short-term memory (LSTM) model with an attention mechanism to predict Kellgren/Lawrence (KL) grade for knee osteoarthritis patients. The attention scores reveal a time-associated impact of different variables on KL grades. We also employed a fast causal inference (FCI) algorithm to estimate the causal relation of key variables, which will aid in clinical interpretability. Based on the clinical information of current visits, we accurately predicted the KL grade of the patient's next visits with 90% accuracy. We found that joint space narrowing was a major contributor to KOA progression. Furthermore, our causal structure model indicated that knee alignments may lead to joint space narrowing, while symptoms (swelling, grinding, catching, and limited mobility) have little impact on KOA progression. This study evaluated a broad spectrum of potential risk factors from clinical data, questionnaires, and radiographic markers that are rarely considered in previous studies. Using our statistical model, providers are able to predict the risk of the future progression of KOA, which will provide a basis for selecting proper interventions, such as proceeding to joint arthroplasty for patients. Our causal model suggests that knee alignment should be considered in the primary treatment and KOA progression was independent of clinical symptoms.

## Introduction

Osteoarthritis (OA) is a common disease in older individuals, and the economic burden of OA rapidly increases with obesity prevalence and aging in the United States. Knee osteoarthritis (KOA) is the most prevalent type of osteoarthritis with around 22.7% (54.4 million) adults diagnosed with arthritis in the United States (1). The main symptoms of knee OA (KOA) are pain, stiffness, and swelling. These symptoms cause inconvenience to everyday life, and some people may even lose their physical ability due to this. Once the symptoms appear, it is hard to be cured since damage to the joints cannot be reserved. There is no effective disease-specific drug for this irreversible degenerative disease currently (2). Medication only helps to relieve OA symptoms, primarily pain. Since OA is a slow-developing disease, it is often undiagnosed until symptoms appear, so the best treatment opportunity is missed. It is estimated that the number of total knee replacement surgery or knee arthroplasty (TKA) will reach 1.26 million by 2030 in the United States (3). Considering the rapid increase of KOA patients, it is important to detect KOA at an early stage and perform intervention treatment before knee condition deteriorates.

The most common standard of quantifying the severity of KOA is the five-grade Kellgren and Lawrence (KL) system (4). This grading system divides KOA into four stages, ranging from 0 to 4. Grade 0 indicates no evidence of KOA and grade 4 indicates severe KOA. Medically, incident radiographic KOA is defined when KL grade is ≥2 (5). A great number of works have been done to identify risk factors for the occurrence of KOA (6, 7). Zhang et al. used a logistic regression model and identified age, sex, body mass index (BMI), occupational risks, injury, and family history of KOA as risk factors (8). However, these studies only identified risk factors for the occurrence of KOA (whether KL ≥ 2), instead of the progression of the disease. There were also some attempts to quantify KOA severity. Du et al. employed support vector machine, random forest, and naive Bayes to predict the progression of KOA from 3-D magnetic resonance (MR) imaging (9). Although these specific models can quantify KOA severity, most attempts were built on image processing and were hard to interpret in the clinical setting. Therefore, our study was designed to build a predictive model with patients' assessment data such as symptoms, questionnaire data, and interpretable image features and identify the key risk factors during the disease progression. Besides the predictive model, we also adopted the causal inference to verify the risk factors identified in predictive models. In order to get higher accuracy, the predictive model may include some unnecessary predictors since it only considers the association between the dependent variable and predictors. However, if physicians take all predictors under consideration in intervention treatment, it may cause overtreatment, because some predictors are not the cause of the disease. In order to eliminate the effect of unknown factors (also called confounders) and avoid unnecessary therapies, we adopted a causal analysis to estimate the causal effect relationship between clinical factors and radiographic markers. The directed graphical causal models (DGCM) can identify the causal relationship instead of association (10). This causal relation is the result of multiple hypothetical experiments by measuring how much an error score will change when the values of a variable are randomly permuted (11). These experimental predictions are computed from the probability distribution. Therefore, a causal relation is defined when variable A and variable B co-vary if we only changed variable A (12). In this study, we used the fast causal inference (FCI) algorithm (12) for causal inference. FCI is designed to test conditional independence. It first generates a complete undirected graph and then deletes recursively edges based on the conditional independence decisions.

The purpose of this study is to use short-term data to predict long-term KOA progression by inputting observed time series into an attention–long short-term memory (LSTM) model and outputting the likelihood of patients' KL grade. Additionally, we built a causal model that evaluates the causal structure of potential predictors and identified the primary contributors to KOA progression and pain progression. To characterize the OA progression, we used 5-year data from the Osteoarthritis Initiative (OAI), specifically patients' clinical assessment data such as symptoms, questionnaire data, and interpretable radiographic image features.

## Materials and Methods

### Study Population

The OAI is a multicenter observational cohort study with longitudinal clinical and image data. This database includes MRI/CT imaging data, genotyping data, and clinical data for evaluating potential biomarkers and characterizing OA incidence and progression. Individuals from this study were between the ages of 45 and 79 and were at high risk for KOA. We selected patients with at least five visits over the 5-year longitudinal study and excluded patients who underwent knee replacement surgery. In total, 518 patients were included in our study. Among these patients, 394 patients remained in the same level of KL while the knee conditions for other 124 patients had worsen during the 5-year following-up period. We used predictive mean value to impute the missing values. For patients with OA in both knees, we selected the knee with a higher KL grade. We also extracted data from the Cerner Health Facts database to cross-validate our predictive model. This database contains clinical health records from over 500 health care centers across the United States. This clinical dataset uses ICD-9/10 diagnosis codes instead of KL grades.

### Predictors

To capture the full picture of disease progression, we extracted clinical data, questionnaires, and radiographic markers from the OAI. In order to assess the functional status of patients, we adopted the Western Ontario and McMaster Universities Osteoarthritis Index (WOMAC). The WOMAC questionnaire evaluates the level of pain, stiffness, and function of the knee. The American College of Rheumatology considers this questionnaire as the gold standard for KOA functional status (13). For the clinical data, we chose patients' characteristics that could potentially predict future KOA, such as age, BMI, and measurements of physical ability. The radiographic markers, such as joint space width and knee alignment (the hip–knee–ankle angle), were extracted from X-ray. The minimal joint space width (mJSW) has been considered as a proxy for cartilage thickness (14). We first analyzed different independent predictors of KL grade in a multivariate logistic regression model, which is assumed as the basic standard in KOA analysis. BMI and age were analyzed as a categorical variable. BMI is divided into four groups: underweight (BMI 10–19), normal (BMI 20–26), overweight (BMI 26–34), and obese (35+). Age was divided into 5-year intervals, namely 50–54, 55–59, 60–64, 65–69, 70–74, and 75–79. We set the normal BMI and age between 45 and 49 as the reference for the odds ratio. For the questionnaire, we marked “no symptom” as the reference and the highest score as the worst condition of the individual in that question. Table 1 shows the independent predictors of KL grade in a multivariate logistic regression model.

**Table 1:** Multivariable logistic regression model demonstrating independent predictors of KL >1.

**Predictors**	**Odds ratio**	**[95% conf. interval]**		***p* > *z***	***p* (>Chi)**
**JSPAINPRG**	JSL and pain progressor				0.3391265
1	0.63	0.39	1.01	0.054881	
**LKALNMT**	1.02	0.95	1.1	0.623373	0.3143121
**RKALNMT**	0.96	0.89	1.03	0.263646	0.56715
**JSONLYPRG**	JSL only progressor				0.287
1	0.5	0.29	0.87	0.013476	
**PAINONLYPRG**	Pain only progressor				0.3808985
1	0.73	0.44	1.21	0.212488	
**XRJSM**	4.1	2.9	5.86	<0.001	<0.001
**XRJSL**	1.04	4.38	2.67	0.934219	0.5478206
**MCMJSW**	0.84	6.83	1.04	0.11842	0.19541
**BMI**	Body mass index				<0.001
Overweight	3.35	2.01	5.58	<0.001	
Obese	5.44	3.23	9.2	<0.001	
Morbidly obese	13.81	3.49	93.9	0.001	
**WOMKP**	1.16	1.03	1.3	0.012959	<0.001
**WOMADL**	0.97	0.94	1.01	0.174	0.9597197
**P02KPN**	Either knee pain, aching or stiffness: any, in the past 12 months				<0.001
1	0.2	0.08	0.43	<0.001	
**P01KPACT30**	Whether either knee, limit activities due to pain, aching or stiffness, past 30 days				0.490686
1	0.89	0.6	1.33	0.582108	
**SF2**	How much health limit involvement in moderate activities (e.g., moving a table, pushing a vacuum cleaner.)				0.1215677
1: Yes, limited	Ref				
2: Limit a little bit	0.32	0.1	0.87	0.03652	
3: Not limited at all	0.57	0.18	1.49	0.284351	
**WSRKN1**	Right knee stiffness: in the morning, the last 7 days				0.151677
1	0.6	0.37	0.95	0.028428	
2	0.5	0.25	0.97	0.039967	
3	0.55	0.16	2.11	0.353752	
**WSRKN2**	Right knee stiffness: later in the day, the last 7 days				0.8830752
1	0.96	0.59	1.58	0.85981	
2	1	0.49	2.07	0.989272	
3	0.93	0.24	4.21	0.913856	
**KSXRKN1**	Right knee symptoms: swelling, the last 7 days				<0.001
1	2.1	1	4.88	0.063434	
2	2.06	0.89	5.21	0.105648	
3	2.57	0.66	13.29	0.206253	
4	0.67	0.22	2.3	0.495412	
**KSXRKN2**	Right knee symptoms: feel grinding, hear clicking or any other type of noise when knee moves, the last 7 days				0.1097449
1	1.8	0.96	3.52	0.075166	
2	1.49	0.88	2.57	0.141885	
3	2.28	0.98	5.63	0.062791	
4	2.52	0.77	9.27	0.141467	
**KSXRKN3**	Right knee symptoms: knee catch or hang up when moving, the last 7 days				0.0368252
1	2.9	1.38	6.8	0.008418	
2	1.62	0.72	3.86	0.256756	
3	0.93	0.23	4.88	0.920198	
**KSXRKN4**	Right knee symptoms: straighten knee fully, the last 7 days				0.1337019
1	3.86	1.21	14.23	0.029485	
2	6.09	0.85	132.68	0.130066	
**KSXRKN5**	Right knee symptoms: bend knee fully, the last 7 days				0.790488
1	0.64	0.28	1.55	0.307365	
2	0.6	0.18	2.44	0.433231	
3	0.33	0.08	1.52	0.124832	
4	0.32	0.08	1.46	0.114924	
**KSXLKN1**	Left knee symptoms: swelling, the last 7 days				0.1417692
1	2.32	1.04	5.73	0.050612	
2	1.35	0.63	3.13	0.460365	
3	3.93	0.87	30.51	0.117698	
4	2.67	0.69	14.19	0.193381	
**KSXLKN2**	Left knee symptoms: feel grinding, hear clicking or any other type of noise when knee moves, the last 7 days				0.8641942
1	0.84	0.45	1.61	0.58539	
2	0.73	0.42	1.26	0.251584	
3	0.82	0.35	2.01	0.649514	
4	0.76	0.24	2.61	0.651474	
**KSXLKN3**	Left knee symptoms: knee catch or hang up when moving, the last 7 days				0.7839969
1	0.97	0.49	1.99	0.924445	
2	1.1	0.5	2.57	0.81326	
3	0.33	0.064	2.62	0.22145	
**KSXLKN4**	Left knee symptoms: straighten knee fully, the last 7 days				0.1494063
1	0.2	0.07	0.59	0.00364	
2	0.44	0.08	3.62	0.380026	
3	0.04	0.005	0.49	0.006329	
**KSXLKN5**	Left knee symptoms: bend knee fully, the last 7 days				0.0377456
1	3.45	1.18	11.47	0.03219	
2	3.66	1.15	13.96	0.039426	
3	7.66	1.31	70.22	0.039477	
4	5.76	1.17	48.2	0.057999	
**Age**					<0.001
50–54	1.62	0.85	3.17	0.161995	
55–59	3	1.47	6.1	0.002337	
60–64	2.09	1.02	4.21	0.039225	
65–69	3.23	1.51	6.9	0.00243	
70–74	5.61	2.43	13.21	<0.001	
75–79	2.32	1.02	5.29	0.044134	

### Predictive Model

The traditional prediction methods based on time series primarily comprise the autoregressive integrated moving average model (ARIMA), hidden Markov model (HMM), and recurrent neural networks (RNN) (15). However, ARIMA models are usually applied where data shows evidence of non-stationarity and suitable for numerical sequence (16). Since we included some questionnaires as inputs, ARIMA is not a good option. A Markov model is a useful tool to characterize the moving process where an individual transits among multiple states. In this study, we used Markov processes to estimate KOA disease stage transition probabilities. However, one limitation of the Markov model is that it assumes that the future state only depends on the current state (17). Unlike the Markov model, RNN allows the future states to depend on all past states (18).

RNN is a special neural network which could efficiently pass forward information to the next cell at each point (19). For each hidden layer at time point *t*, it not only includes the input layer at time point *t* but also considers the output of the hidden layer at the previous time point *t*−1(20). Although RNN did a very good job in dynamically combining the sequential information based on its internal recurrence (21), RNN may have some issues of gradient vanishing when dealing with long-term data. Therefore, an advanced type of RNN named LSTM was designed to solve the gradient disappearing (22). Unlike other RNNs, LSTM introduced a forget gate (22) to decide whether to keep or drop a cell state based on the previous hidden state and the current input variables. Another important component of LSTM is cell state which is used to control whether to add or remove the information in the previous cell state. It has been proven that LSTM did a better job in long-term time series data (23).

The attention mechanism focuses on certain time points in the time series when processing the data. For example, BMI is very important in the early stage of the disease, while joint space width is more important in the late stage. It allows the model to pay more attention to the most important time point based on what it has learned so far. We adopted the attention mechanism from Nauta's repository and calculated the context vector as a weighted sum of each input vector instead of each time series so that an attention vector learns weights corresponding to input features (11). The attention mechanism assigns a different weight to different variables based on its ability to forecast. All initial attention scores are set to one and updated in every training epoch.

### Causal Inference

In this study, we used FCI with directed acyclic graphs (DAGs). DAGs are commonly used to represent causal relationships, where vertices denote variables and the edges represent causal relationships between variables. The PC algorithm uses statistical tests to find conditional independence and constructs the structure of DAGs based on the results (12). The FCI algorithm is a generalization of the PC algorithm, except that it allows the existence of confounder variables (12). The FCI algorithm is able to detect a Markov equivalence class of DAGs with latent variables based on conditional independence information from the observed variables (12).

There are two important structures in FCI: the “V” structure and the “Y” structure (24). The “V” structure is defined when A and C are independent but dependent conditionally on B, marked as A→B←C. The “Y” structure is defined when A and C are independent of D conditional on B, marked as A→B←C and B→D. The FCI starts with a complete, undirected graph and removes recursively edges based on conditional independence decisions. After finding the skeleton of DAG, edges are oriented by identifying the “V” and “Y” structures, and further orientation rules given by Zhang (25) are applied.

### Validation and Data Integration

**Figure 1:**
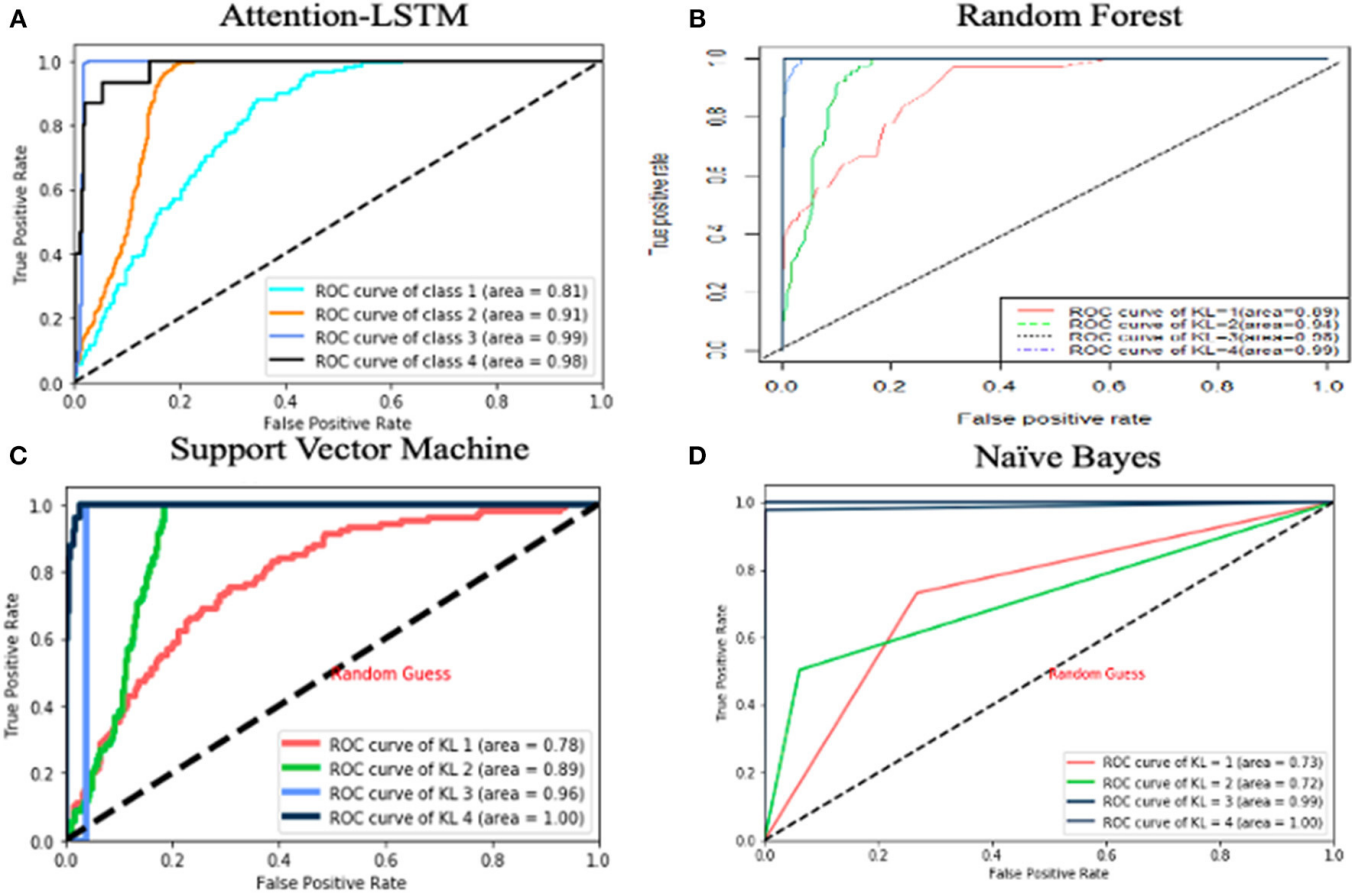
AUC curve for predictive models. The x-axis represents sensitivity and the y axis represents specificity.

We used 10-fold cross-validation for performance evaluation and compared true KL grade vs. the KL grade predicted. Figure 1 reports areas under the receiving operating characteristic curves (ROC). The *x*-axis presents sensitivity (true-positive rate) and the *y*-axis represents specificity (false-positive rate). The area under the curve is defined as AUC, which is a standard of performance of classification. The higher the AUC is, the better the classifier.

One limitation of the OAI dataset is that not every hospital measures KOA-specific features. In order to include these clinical indicators, we chose 30 clinical features from the Cerner database, which are commonly used for KOA diagnosis, but not included in the OAI, such as pulse popliteal of the knee. Previous studies identified age and BMI as the most significant risk factors in the development of KOA (26); therefore, we assumed that patients may have similar clinical indicators with those who shared the same BMI and age. Under this assumption, we used values from the Cerner dataset to estimate the values of variables that are missing in the OAI dataset. For each patient in the OAI dataset, we extracted clinical features from age- and BMI-matched patients from the Cerner database. OAI patients who are age- and BMI-matched with only one patient from the Cerner dataset are directly assigned the matched patient's Cerner clinical values. For patients who matched with multiple patients from the Cerner dataset, we took the average of matched patients and assigned averages to corresponding OAI patients. However, the prediction accuracy of LSTM after this imputation reduced to 85%. Therefore, we applied autoencoder and principal components analysis (PCA) as the denoising feature extractor. The performance of autoencoder is better than PCA and achieved a prediction accuracy of 93% in the dataset combined with the OAI and Cerner data.

## Results

### Characterizing Disease Progression

Before we built the predictive model, we used logistic regression for feature selection. Compared with the other predictive models, such analysis can avoid confounding effects by considering the association of all variables (27). The result of multivariable logistic regression is presented in Table 1. Odds ratio (OR) is the constant effect of a predictor on the occurrence of outcome. Susceptible risk factors, including BMI and age, were associated independently with increased risk of KOA severity. Compared with normal BMI (18.5–24.9), BMI was associated with increased likelihood of KOA severity and the odds of KOA severity increase as BMI increases (overweight: OR: 3.35; 95% CI: 2.01–5.58, *p* < 0.001; obese: OR: 5.44; 95% CI: 3.23–9.20, *p* < 0.001; morbidly obese: OR: 13.81; 95% CI: 3.49–93.9, *p* = 0.001). Age is also a risk factor for KOA. The OR for people between ages 70 and 74 has the highest OR (OR: 5.61; 95% CI: 2.43–13.21, *p* < 0.001). Susceptible joint factors, including joint space narrowing (XRJSM) (OR: 4.10; 95% CI: 2.9–5.86, *p* < 0.001) and knee pain, aching, or stiffness in the past 12 months (P02KPN) (OR: 0.2; 95% CI: 0.08–0.43, *p* < 0.001), were associated with increased risk of occurrence of KOA independently. Swelling in the knee (KSXRKN1) was associated with successively increased odds of occurrence of KOA. Based on the result, we removed three variables from the predictor list for the predictive models, including right knee stiffness status later in the day, the ability to bend right knee fully in the last 7 days, and whether left knee feels grinding.

### Predicting Disease Progression With Attention–LSTM

We investigated the performance of LSTM algorithms in predicting KL grade using clinical data spanning 1 year. The predicted accuracy of LSTM achieved 90%. We compared the LSTM model against the previous models, namely, random forest, support vector machine, and naive Bayes. Random forest (RF) constructs a set of multiple decision trees on data and then averages the prediction from each of them. Compared with a single decision tree, RF reduces the chance of overfitting. Support vector machine (SVM) adopts kernels, which transform a lower dimensional input space into a higher dimensional space. This conversion made SVM more flexible and accurate. Naive Bayes is another classification technology based on Bayes' theory, which assumes that all the predictors are independent to each other. Our LSTM model performs better than RF, SVM, and naive Bayes in predicting KL grade. ROC curves are shown in Figure 1. A value of 0.5 in AUC represents as a random guessing and a value higher than 0.8 is considered quite good. Based on the results, LSTM and RF both did a good job in KL classification. The AUC value of LSTM is higher than that of SVM and naive Bayes for all KL grades, and LSTM has better performance than random forest for KL = 3/4. In other words, random forest did a better job in diagnosing KOA, while LSTM did a better job in predicting KOA severity.

**Figure 2:**
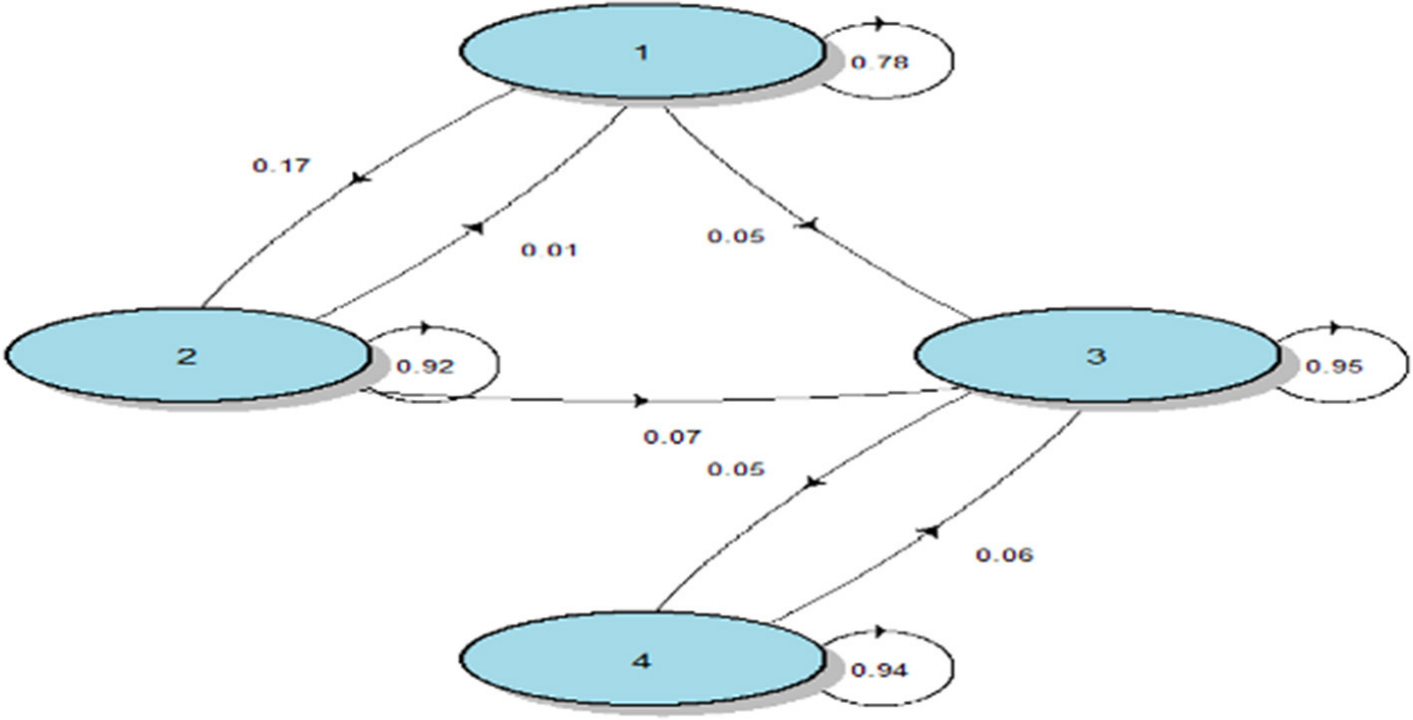
Markov model for KOA progression. Rates of transition between four stages of OA. The number 1, 2, 3, 4 represent four stages of KOA. The arrows are the transitions rates between these states.

To better describe disease progression, we consider Markov states as disease stages. The Markov chain result is shown in Figure 2. For example, the patients at high risk (KL = 1) have 17% chance to move to the next stage (KL = 2) and 78% chance to maintain their KL grade after the 1-year follow-up visit. It should be noted that patients diagnosed with knee osteoarthritis (KL ≥ 2) are more likely to remain at this stage and very less likely to revert back to previous disease states.

### Importance Analysis of Variables

**Figure 3:**
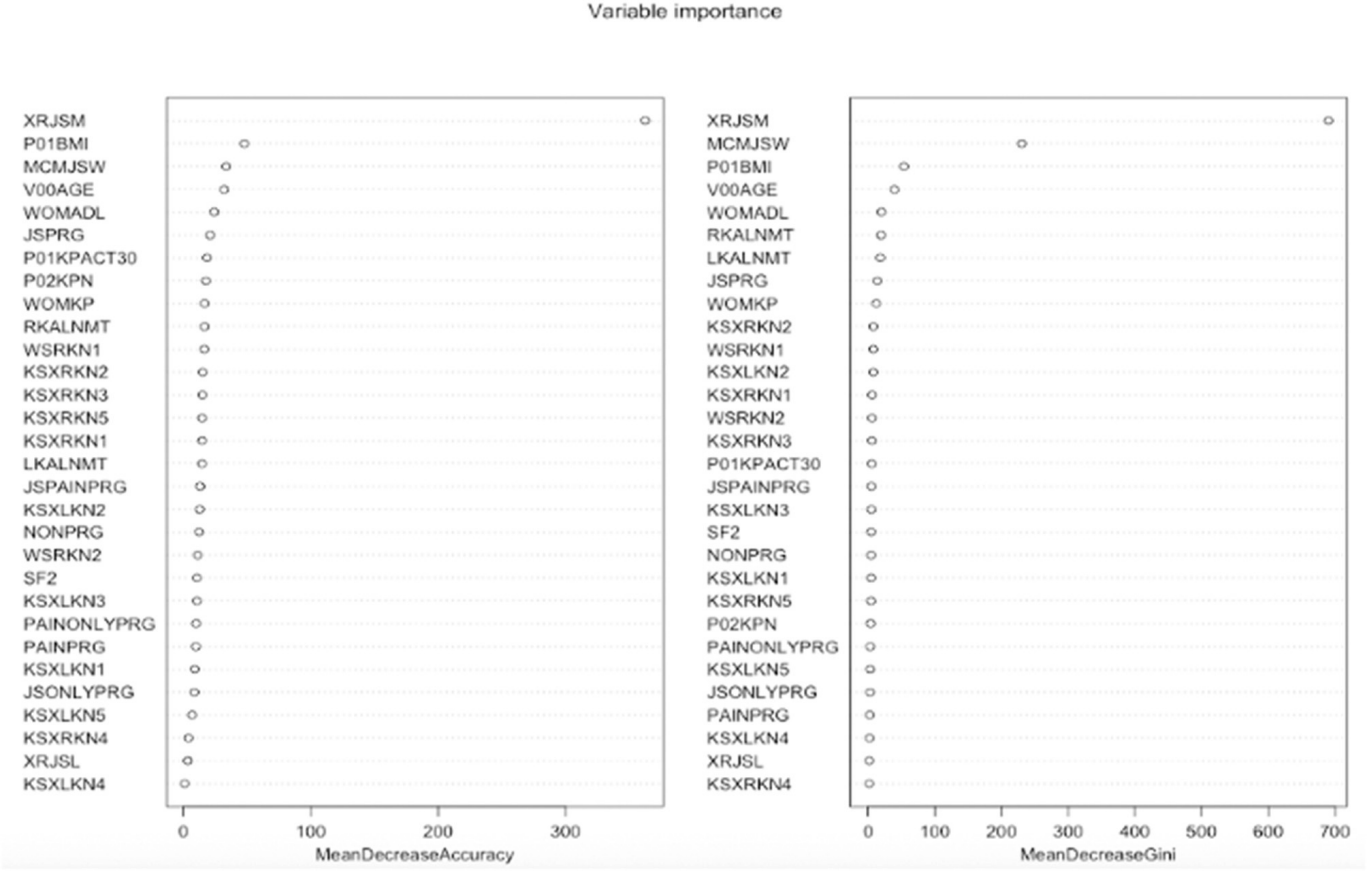
Features importance of random forest. The left figure is mean decrease in accuracy which measures misclassification of removing the given variable. The right figure is mean decrease in Gini which measures the average gain by splitting the given variable.

Figure 3 graphically depicts the important indexes of random forest. We used the mean decrease in accuracy index to evaluate the importance of variables in classification. It shows that the variable “XRJSM” (joint space narrowing) stands out among all the variables with the largest mean decrease in accuracy. Variables “P01BMI” (BMI), “MCMJSW” (medial minimum joint space width), “V00AGE” (age), and “WOMADL” (WOMAC disability score) are also relatively important for predicting KOA severity based on the indexes of variable importance.

**Figure 4:**
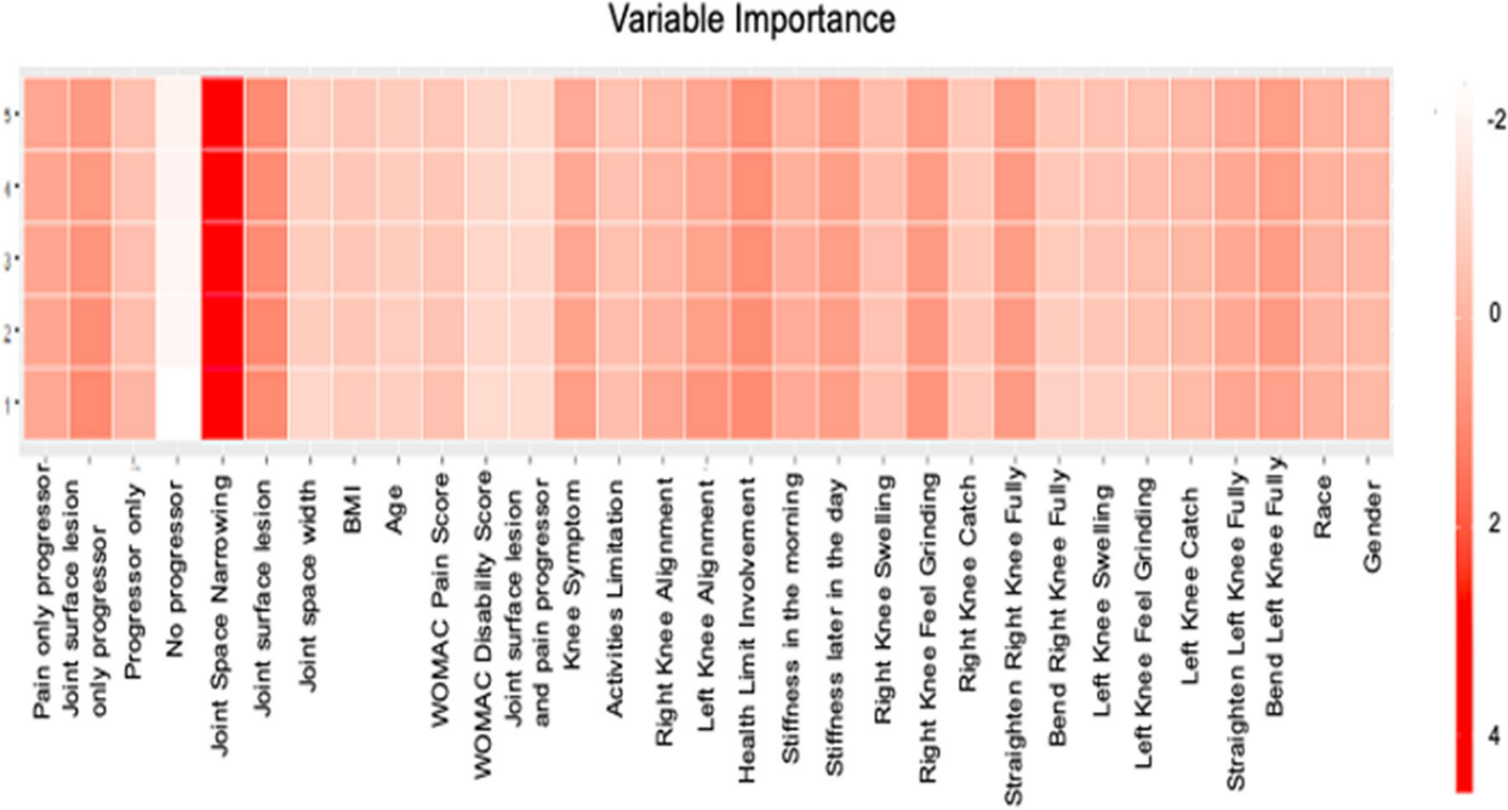
Features Importance of LSTM. The x-axis represents the name of variable and the y axis represents the number of visit. The color represents the attention score.

The attention maps in Figure 4 demonstrate how attention scores can identify the dynamics. Although it was hard to distinguish which visit would have the model attended the most, XRJSM (OARSI joint space narrowing grade) was significantly important in predicting KL grade. The attention mechanism identified that joint space narrowing leads to the worsening status of KOA, which causes pain. The finding also explained why joint space narrowing is the most important factor in predicting KL progression in the LSTM model.

### Causal Relationship of Variables

**Figure 5:**
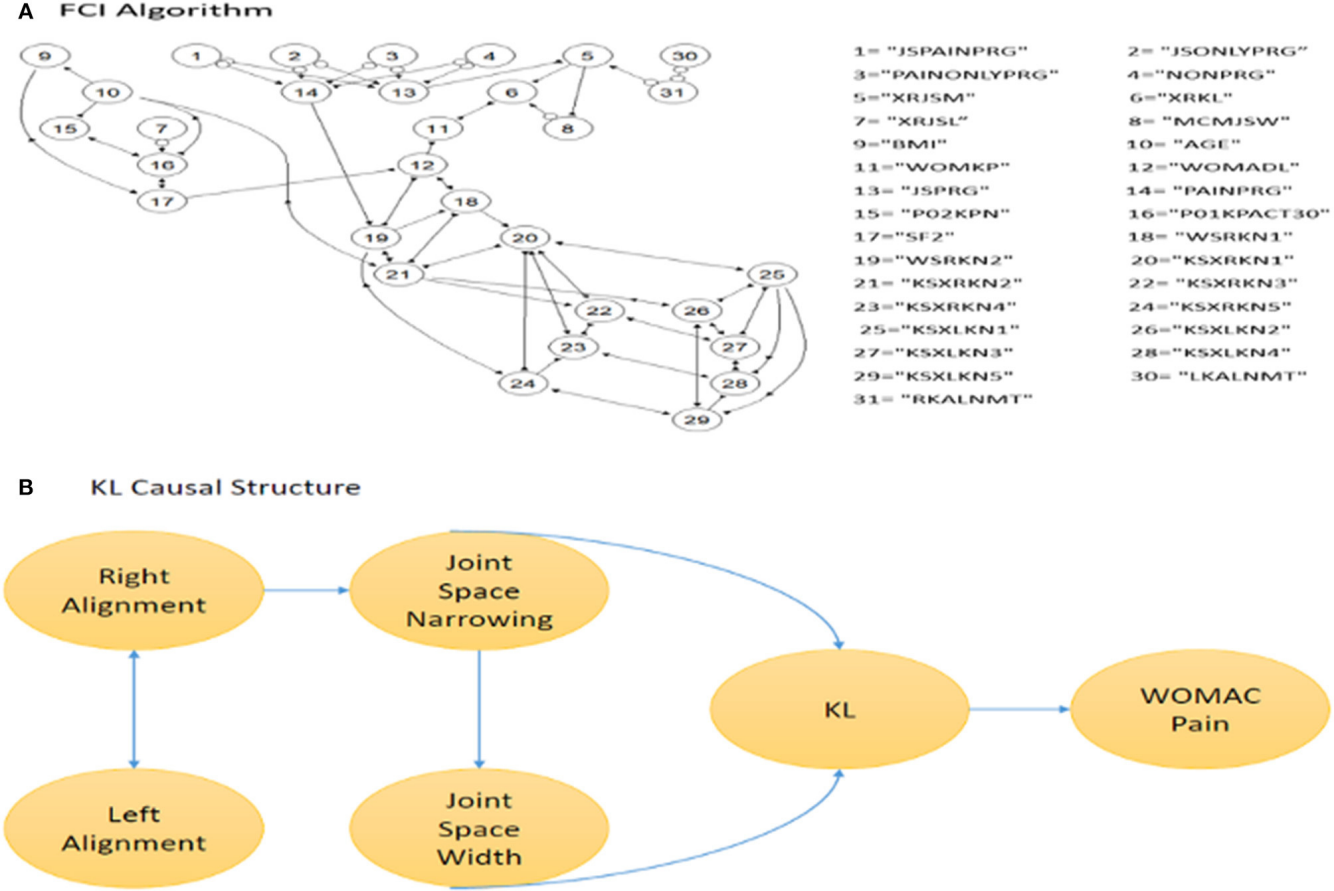
Causal Inference. **(A)** the result of FCI in DAGs. The number denotes the variable and the edges represent the causal relationship. **(B)** is snapshot of a small part of **(A)**.

To understand the dependency and independency of important features in predicting KL progression, we employed FCI. We found that misalignments of the left and right knee are the reason for joint space narrowing. The angles of tibiofemoral and patellofemoral joints affected the alignment of the knees and caused joint space narrowing. This is consistent with previous reports that various knee alignment is associated with the radiographic measures of KOA severity (28). The FCI output is presented in Figure 5. The bidirected edge between left alignment and right alignment indicates that there exists at least one unmeasured confounder of left alignment and right alignment. The “o” symbols at alignment and joint space narrowing (JSM) indicate that it is difficult to distinguish whether the connection between alignment and JSM connection is a directed edge or an unmeasured confounder. This result also eliminated some unnecessary predictors from the LSTM model. Although the symptoms, such as swelling, feel grinding, knee catch, straighten knee full, and bend knee full, may be good predictors for KL grade, they are not the reason causing KOA progression. Symptoms seem to be a good predictor of pain progression. Using FCI, we find that the change in joint space is the real reason for disease progression.

## Discussion

KOA is a slowly progressive disease with irreversible joint damage. With obesity prevalence and aging in the United States, KOA becomes the most frequent disease. However, an accurate predictive model with diagnostic criteria is still unavailable. Therefore, the goal of this study was to develop a mathematic model to predict OA severity and the key risk factors associated with disease progression. In this study, we used logistic regression for feature selection. After removing the irrelevant variables, we employed the attention–LSTM predictive model to predict OA severity and compared this approach against the existing methods. RNN, especially LSTM, did a good job in modeling long-term dependency in time series data. The AUC values of LSTM for KL = 1, 2, 3, and 4 are 0.81, 0.91, 0.99, and 0.98, respectively. The attention mechanism allows us dynamically to detect the feature importance across multiple time steps for predicting KOA progression. Meanwhile, the attention scores extracted from the attention mechanism would help to discover the direction of causal relationship. Finally, we use causal inference to interpret the inside connection of variables. To validate our predictive model, we used the OAI database in conjunction with the Cerner Health Facts database. Our experiments showed that the attention–LSTM with the scaled autoencoder resulted in 3% increased accuracy (from 90 to 93%). Hence, this method can be a promising tool for patients and doctors to prescreen for possible osteoarthritis to prevent deterioration of OA, thereby supporting for clinical decision-making.

Our attention–LSTM model not only reliably and accurately predicts KL grade and progression but also identifies the key factors among different time points. Using our model, clinicians can predict the possible KOA progression of patients and take preventative measures in advance. The attention mechanism dynamically shows the importance of variables at different disease stages and indicates that joint space narrowing is the primary factor in KOA progression at all time points. This method will provide strong support in clinical decision-making, including diagnosis, appropriate treatments, and preventive care. Using causal inference analysis, we identified the real cause of joint space narrowing and pain. They are misalignments of tibiofemoral and patellofemoral joints. Current primary preventive intervention is limited to weight loss (29). This finding recommended another possible intervention in clinical practice. The causal discovery also helped providers to avoid overtreatment. For example, treatment for symptoms such as swelling, grinding, catching, and inability to straighten or fully flex the knee may not be able to prevent the worsening status of knee OA. Our work had two major clinical contributions. (1) We evaluated a broad spectrum of potential risk factors (clinical variables such as age, BMI, clinical symptoms, WOMAC questionnaires, and image measurements from X-rays) and investigated the performance of RNN algorithms in predicting KL grade. By using Markov hidden states as the disease stages, we have gained more knowledge about the stage transition, which enables a deeper understanding of the temporal progression of OA. (2) Considering the existence of hidden confounding variables, we built a causal structure of candidate risk factors and identified the preventable factors for treatment.

This study has several limitations to be considered. First, the research target of the OAI is individuals at high risk, with expected overweight and aging population. Thus, our model may not be applicable to the general population. The continuing work will focus on testing its generalizability of the models to different populations. To improve the expansion ability, we would consider transfer learning and generative-adversarial-network-based method in further studies. Second, when interpreting the findings, we found that symptoms of right knee and left knee have different impacts on KOA progression. This finding required some external validation. An additional limitation of this study is the small sample size. We have included prominent OA symptoms such as swelling joint pain, stiffness, and bending (30) in this study. However, we were unable to adjust for factors that may affect long-term outcomes, such as other symptomatic joint diseases.

In conclusion, we used attention scores in LSTM to describe feature importance at different time points and compared our model with previous works. In addition, we used causal inference to identify the key diagnostic criteria in disease progression. Our study has illustrated that clinical symptoms are important in predicting disease severity but may not be essential in disease progression. With the help of causal inference, LSTM is a better tool to help physicians in decision-making.

## Data Availability Statement

The original contributions presented in this study are included in the article/supplementary materials, further inquiries can be directed to the corresponding author/s.

## Author Contributions

YW: data preparation, predicting models, causal inference, statistical analysis, and manuscript preparation. LY: study design and manuscript editing. JC: manuscript editing. LL: manuscript review. PN: clinical support and manuscript editing. XZ: study design, manuscript editing, and responsible for the integrity of this work. WZ: final version manuscript review. YZ: data extract and data management from Cerner Heath Facts database. HX: responsible for Cerner Health Facts and manuscript review. All authors contributed to the article and approved the submitted version.

## Conflict of Interest

The authors declare that the research was conducted in the absence of any commercial or financial relationships that could be construed as a potential conflict of interest.

## References

[1] Lawrence RC Felson DT Helmick CG Arnold LM Choi H Deyo RA Estimates of the prevalence of arthritis other rheumatic conditions in the United States. Part II. Arthritis Rheum. (2008) 58:26–35. 10.1002/art.23176 18163497 PMC3266664

[2] Losina E Daigle ME Suter LG Hunter DJ Solomon DH Walensky RP. Disease-modifying drugs for knee osteoarthritis: can they be cost-effective? Osteoarthritis Cartilage. (2013) 21:655–67. 10.1016/j.joca.2013.01.016 23380251 PMC3670115

[3] Sloan M Premkumar A Sheth NP. Projected volume of primary total joint arthroplasty in the U.S., 2014 to 2030. JBJS. (2018) 100:1455–60. 10.2106/JBJS.17.01617 30180053

[4] Kellgren JH Lawrence JS. Radiological assessment of osteo-arthrosis. Ann Rheum Dis. (1957) 16:494–502. 10.1136/ard.16.4.494 13498604 PMC1006995

[5] Kohn MD Sassoon AA Fernando ND. Classifications in brief: kellgren-lawrence classification of osteoarthritis. Clin Orthop Relat Res. (2016) 474:1886–93. 10.1007/s11999-016-4732-4 26872913 PMC4925407

[6] Sharma L Hochberg M Nevitt M Guermazi A Roemer F Crema MD. Knee tissue lesions and prediction of incident knee osteoarthritis over 7 years in a cohort of persons at higher risk. Osteoarthritis Cartilage. (2017) 25:1068–75. 10.1016/j.joca.2017.02.788 28232012 PMC5466844

[7] Neogi T Zhang Y. Epidemiology of osteoarthritis. Rheum Dis Clin North Am. (2013) 39:1–9. 10.1016/j.rdc.2012.10.004 23312408 PMC3545412

[8] Zhang W McWilliams DF Ingham SL Doherty SA Muthuri S Muir KR. Nottingham knee osteoarthritis risk prediction models. Ann Rheum Dis. (2011) 70:1599. 10.1136/ard.2011.149807 21613308

[9] Du Y Almajalid R Shan J Zhang M. A novel method to predict knee osteoarthritis progression on MRI using machine learning methods. IEEE Trans NanoBiosci. (2018) 17:228–36. 10.1109/TNB.2018.2840082 29994316

[10] Glymour C Zhang K Spirtes P. Review of causal discovery methods based on graphical models. Front Genet. (2019) 10:524. 10.3389/fgene.2019.00524 31214249 PMC6558187

[11] Nauta M Bucur D Seifert C Causal discovery with attention-based convolutional neural networks. Mach Learn Knowl Extr. (2019) 1:312–40. 10.3390/make1010019

[12] Spirtes P Glymour C Scheines R Causation, Prediction, and Search. Cambridge, MA: The MIT Press (2001). 10.7551/mitpress/1754.001.0001

[13] Fernandes MTP Fernandes KBP Marquez AS Cólus IMS Souza MF Santos JPM. Association of interleukin-6 gene polymorphism (rs1800796) with severity and functional status of osteoarthritis in elderly individuals. Cytokine. (2015) 75:316–20. 10.1016/j.cyto.2015.07.020 26233477

[14] Castaño-Betancourt MC Evans DS Ramos YFM Boer CG Metrustry S Liu Y. Novel genetic variants for cartilage thickness and hip osteoarthritis. PLoS Genet. (2016) 12:e1006260. 10.1371/journal.pgen.1006260 27701424 PMC5049763

[15] Almqvist O A comparative Study Between Algorithms for Time Series Forecasting on Customer Prediction: An Investigation Into the Performance of ARIMA, RNN, LSTM, TCN and HMM. (2019). Available online at: http://www.diva-portal.org/smash/get/diva2:1321224/FULLTEXT01.pdf

[16] Boshnakov G Introduction to Time Series Analysis Forecasting, 2nd Edition, Wiley Series in Probability Statistics, by Douglas C.Montgomery, Cheryl L.Jennings MuratKulahci (eds). Published by John Wiley Sons, Hoboken, et al. Total number of pag: INTRODUCTION TO TIME SERIES ANALYSIS AND FORECASTING, 2ND EDITION, WILEY SERIES IN PROBABILITY AND STATISTICS, by Douglas Montgomery C, Cheryl L. Jen. J Time Ser Anal. (2016) 37:864 10.1111/jtsa.12203

[17] Awad M Khanna R Hidden Markov Model, Efficient Learning Machines: Theories, Concepts, and Applications for Engineers and System Designers, Apress, Berkeley, CA. (2015). p. 81–104. 10.1007/978-1-4302-5990-9_5

[18] Alaa AM van der Schaar M Forecasting Individualized Disease Trajectories Using Interpretable Deep Learning. (2018). Available online at: https://arxiv.org/pdf/1810.10489.pdf

[19] Russell S Norvig P Artificial Intelligence: A Modern Approach. New Jersey, NJ: Prentice Hall Press (2009). Available online at: https://www.cin.ufpe.br/~tfl2/artificial-intelligence-modern-approach.9780131038059.25368.pdf

[20] Graves AJA Generating Sequences With Recurrent Neural Networks. (2013). Available online at: https://arxiv.org/pdf/1308.0850.pdf

[21] Murtagh F Starck J-L Renaud O On neuro-wavelet modeling. Decis Support Syst. (2004) 37:475–84. 10.1016/S0167-9236(03)00092-7

[22] Hochreiter S Schmidhuber JA. Long short-term memory. Neural Comput. (1997) 9:1735–80. 10.1162/neco.1997.9.8.1735 9377276

[23] Palangi H Deng L Shen Y Gao J He X Chen J Deep sentence embedding using long short-term memory networks: analysis and application to information retrieval. IEEE. (2016) 24:694–707. 10.1109/TASLP.2016.2520371

[24] Kalisch M Mächler M Colombo D Maathuis MH Bühlmann P Causal inference using graphical models with the R Package pcalg. J Stat Software. (2012) 47:26 10.18637/jss.v047.i11

[25] Zhang J On the completeness of orientation rules for causal discovery in the presence of latent confounders and selection bias. Artif Intell. (2008) 172:1873–96. 10.1016/j.artint.2008.08.001

[26] Driban JB McAlindon TE Amin M Price LL Eaton CB Davis JE Risk factors can classify individuals who develop accelerated knee osteoarthritis: data from the osteoarthritis initiative. J Orthop Res. (2018) 36:876–80. 10.1002/jor.23675 28776751 PMC5797506

[27] Sperandei S. Understanding logistic regression analysis. Biochem Med (Zagreb). (2014) 24:12–8. 10.11613/BM.2014.003 24627710 PMC3936971

[28] Sharma L Song J Felson DT Cahue S Shamiyeh E Dunlop DD. The role of knee alignment in disease progression and functional decline in knee osteoarthritis. JAMA. (2001) 286:188–95. 10.1001/jama.286.2.188 11448282

[29] Vincent HK Heywood K Connelly J Hurley RW. Obesity and weight loss in the treatment and prevention of osteoarthritis. PM R. (2012) 4:S59–67. 10.1016/j.pmrj.2012.01.005 22632704 PMC3623013

[30] Heidari B. Knee osteoarthritis prevalence, risk factors, pathogenesis features: Part I. Caspian J Intern Med. (2011) 2:205–12. 24024017 PMC3766936

